# Postoperative complication of endoscopic nasopharyngeal multiple cysts resection with skull base osteomyelitis: Case report

**DOI:** 10.1097/MD.0000000000044344

**Published:** 2025-09-12

**Authors:** Wei Wang, Fang Wang, Ping Liu

**Affiliations:** a Department of Otorhinolaryngology, Shaoxing People’s Hospital, Shaoxing, Zhejiang Province, China.

**Keywords:** nasopharyngeal cyst, skull base osteomyelitis

## Abstract

**Rationale:**

Osteomyelitis of the skull following the endoscopic resection of multiple nasopharyngeal cysts is extremely rare. Postoperative headache, in the absence of fever or ear pain, may no be typically present and could be easily overlooked, potentially leading to a delay in diagnosis and treatment.

**Patient concerns:**

In this case report, we present the case of a 57-year-old male patient who developed persistent headache symptoms 1 week after undergoing endoscopic resection of nasopharyngeal cysts under general anesthesia and was diagnosed with skull base osteomyelitis (SBO).

**Diagnoses:**

SBO.

**Interventions:**

After the diagnosis of SBO, the patient was treated with a full course of antibiotics, including 2.0 g ceftriaxone for intravenous infusion for 1 week, 2.5 g of piperacillin sodium and sulbactam for intravenous infusion 12 hours for 2 weeks, and 0.6 g of linezolid tablets for oral administration twice a day for 2 weeks. Under local conditions, endoscopic nasopharyngeal incision and drainage were performed, during which necrotic tissue and pus were removed, and deep tissue secretions were taken.

**Outcomes:**

In the follow-up 6 months at the outpatient clinic after discharge, the patient’s headache improved, no complications occurred, and no symptoms of cranial nerve palsy were observed.

**Lessons:**

Magnetic resonance imaging revealed 3 deep cysts in the nasopharynx. During surgery, when using plasma to resect cysts, it is necessary to avoid excessive exposure or damage to the bone, which would increase the risk of postoperative SBO.

## 1. Introduction

**Figure 1.**
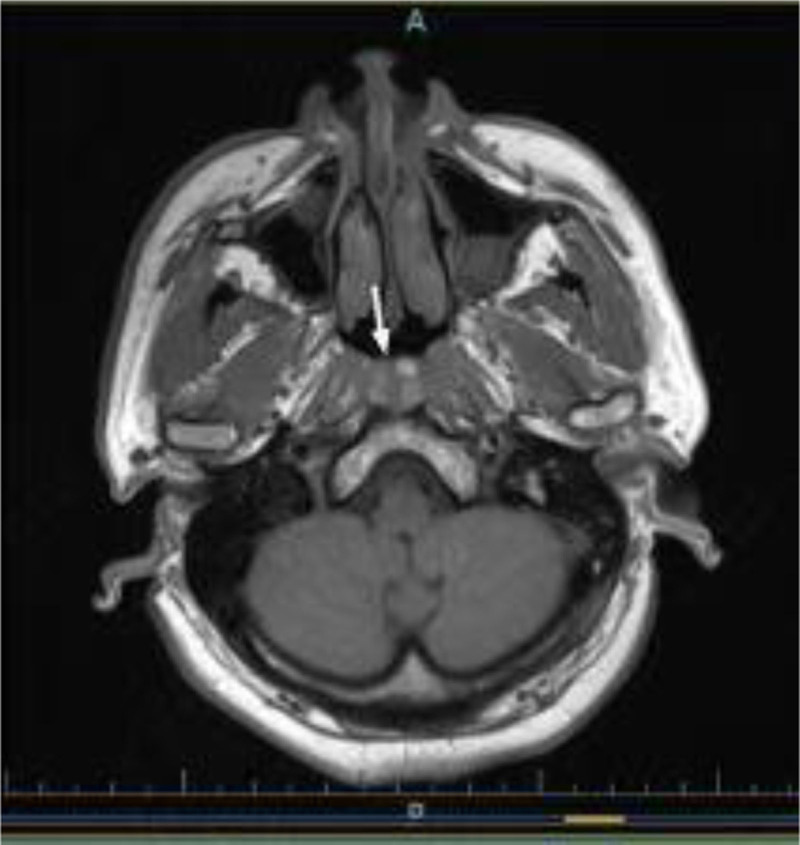
Nasopharyngeal MRI: white arrow indicate 3 nodular lesions with isointense signal. MRI = magnetic resonance imaging.

**Figure 2.**
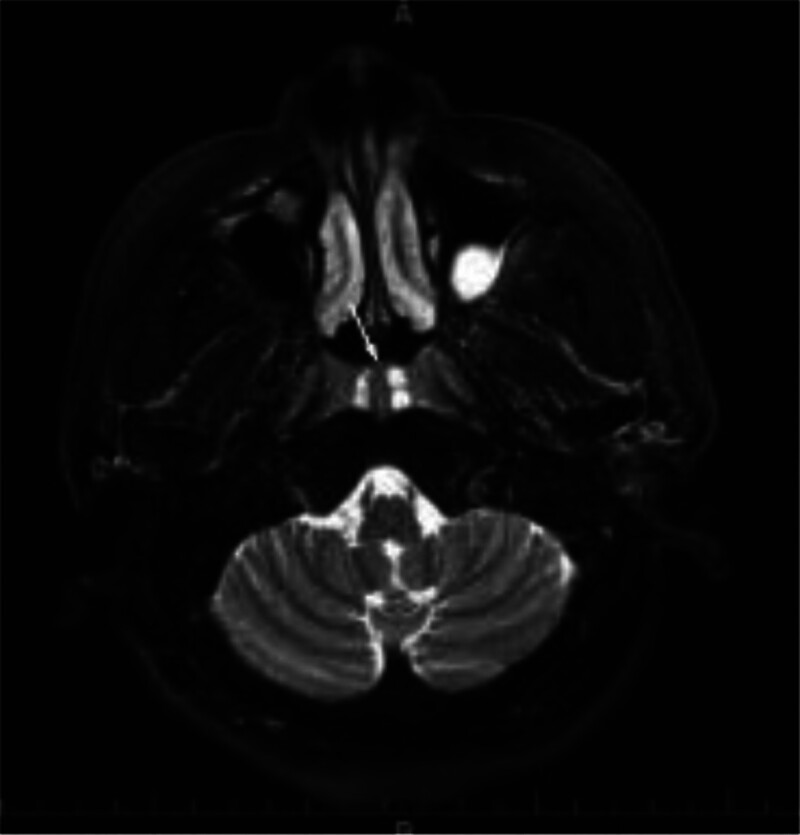
Nasopharyngeal MRI: white arrow indicate 3 nodular lesions with long T2 signal intensity. MRI = magnetic resonance imaging.

**Figure 3.**
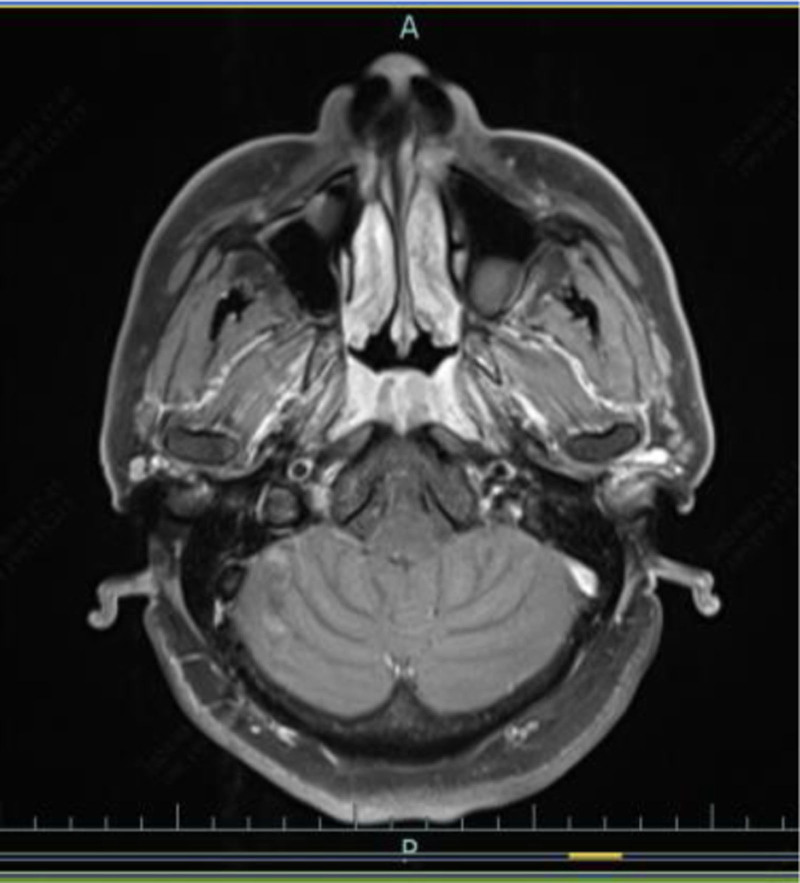
Nasopharyngeal MRI: no post-contrast enhancement is seen in the lesions. MRI = magnetic resonance imaging.

Skull base osteomyelitis (SBO) is a rare and potentially fatal disease^[1]^ that presents with clinical symptoms such as severe headache and ear pain, which can lead to manifestations of cranial nerve involvement, with rapid progression and high mortality (14.3–22%).^[2,3]^ SBO is a severe and rare type of skull base infection, most commonly seen in aged and elderly individuals, and uncommon in those under 25 years of age (>10%), with no significant gender difference.^[4]^ The skull, as a bridge and barrier between the intracranial and extracranial environments, contains many important anatomical structures, such as nasopharynx blood vessels, nerves, and bone tissue. Mortazavi et al^[5]^ classified SBO into 2 major categories (nasal-otogenic and non-nasal-otogenic) based on etiology (iatrogenic, traumatic, hematogenous, and other causes). *Pseudomonas aeruginosa*, *Staphylococcus aureus*, and fungal infections were the most common pathogens.^[6]^ Although the incidence of SBO is not high, it can cause serious harm to life if it is not treated in time in the early stage owing to the deep location of the lesion (Figs. 1–3).

## 2. Case presentation

**Figure 4.**
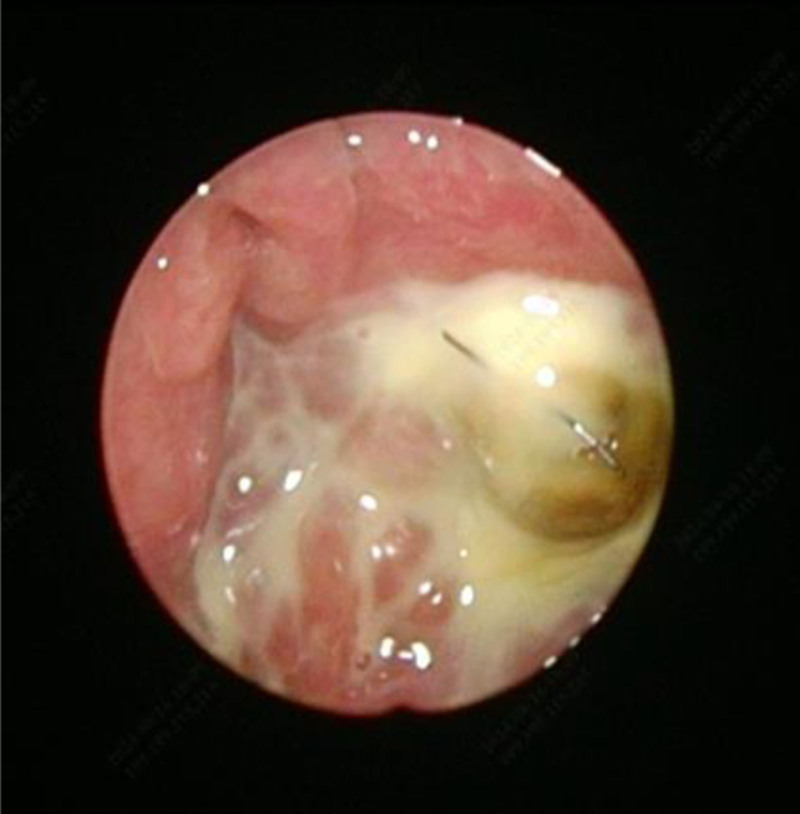
Endoscopic examination of the nose revealed a large amount of purulent secretions in the nasopharynx.

**Figure 5.**
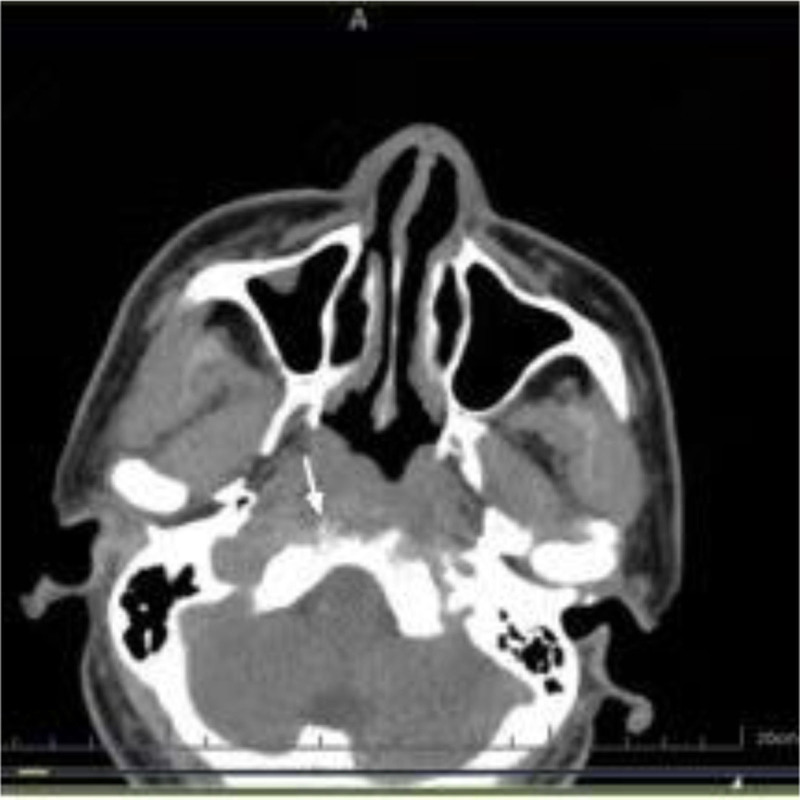
Nasopharyngeal CT: There is thickening of the soft tissue on the right side of the nasopharynx, with localized bone destruction in the right clivus. CT = computed tomography.

**Figure 6.**
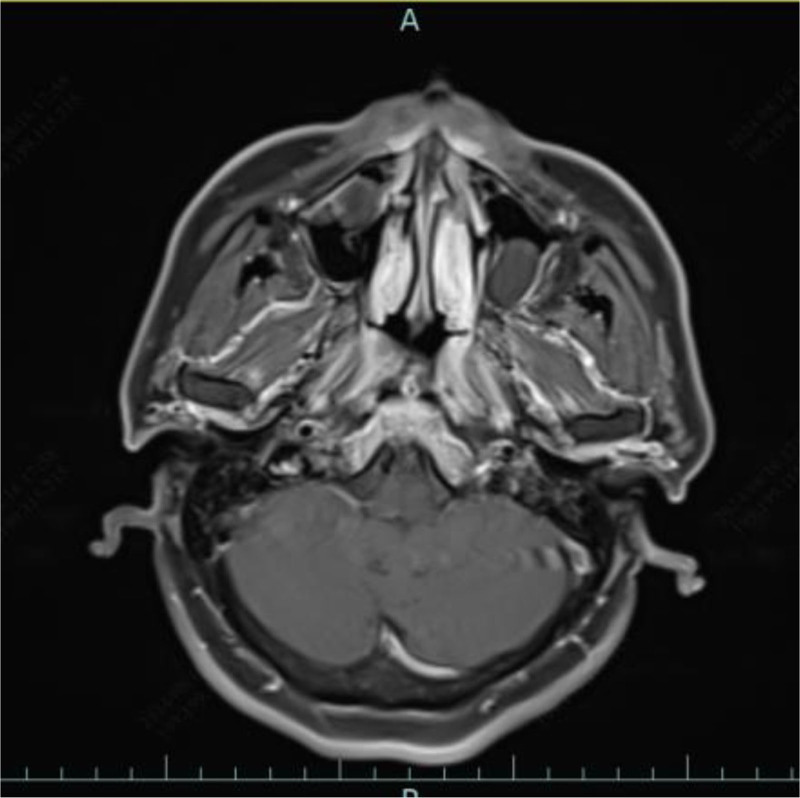
Reexamination of nasopharyngeal enhanced MRI suggested that the nasopharyngeal mucosa was slightly thickened and there was no obvious enhancement. MRI = magnetic resonance imaging.

A 57-year-old man was admitted for day surgery because a mass in the nasopharynx was found during physical examination at 1 week. He had a history of hypertension for 5 years, was taking candesartan ester 80 mg qd for antihypertensive therapy, and his blood pressure was well controlled. He was diagnosed with diabetes more than 2 months ago, and he had not been taking medicine regularly. His blood control was poor, with a glycosylated hemoglobin level of 7.6% and a postprandial blood sugar of 13.5 mmol/L. He underwent endoscopic nasopharyngeal mass resection under general anesthesia at our hospital on May 29, 2024. Cyst-like masses were removed during the operation, and the masses were incised with cystic fluid. Postoperative pathological report: consistent with cysts. A week after the surgery, the patient has persistent headache, mainly in the occipital region, accompanied by pharyngalgia, no fever, chills, etc. This patient has no symptoms of cranial nerve palsy. The outpatient department was given oral cefuroxime axetil tablets 0.25 g for anti-inflammatory treatment, but the patient’s headache did not relieve. On July 5th, a computed tomography scan of the nasopharynx suggested that, after resection of the nasopharyngeal lesion, the nasopharyngeal wall was significantly thickened, the slope bone was destroyed, and inflammation of the sphenoid sinus was observed (Fig. 4). Endoscopic examination of the nose revealed a large amount of purulent secretions in the nasopharynx (Fig. 5). C-reactive protein was 59.03 mg/L. Routine blood test results were normal. Admitted on July 11, 2024, for “osteomyelitis of the skull base,” treated with 2.0 g of ceftriaxone intravenously for anti-inflammatory treatment, and blood sugar was also controlled. The patient’s headache numeric rating scale score changed from 7 to 6 after 1 week of treatment. Change to piperacillin sodium sulbactam 2.5 g intravenous infusion every 12 hours for 11 days. Endoscopic nasopharyngeal incision and drainage were performed under local anesthesia on July 22, during which necrotic tissue and pus were removed, and deep tissue secretions were collected for culture. Secretion culture: Hemolytic Staphylococcus (±±). The sensitivity of the drug was changed by the administration of 0.6 g of linezolid tablets bid for 2 weeks. Reexamination of nasopharyngeal enhanced magnetic resonance imaging (MRI) suggested that the nasopharyngeal mucosa was slightly thickened and there was no obvious enhancement (Fig. 6). Three days after the surgery, the patient’s headache was significantly relieved and he was discharged patient developed serous otitis media in the right ear 3 months later, which improved after 3 weeks of nasal spray steroids. At the outpatient follow-up 6 months after discharge, the patient has a good recovery, no headache, no symptoms of cranial nerve paralysis, no symptoms of ear congestion, etc.

## 3. Discussion

Nasopharyngeal cysts are common benign masses in the nasopharynx, and postoperative infections complicated by skull base osteotomy are extremely rare. Exposing healthy bones to pathogens during surgery may increase the risk of infection.^[7]^ The patient in this case had 3 cysts in the nasopharynx on MRI, which were widely distributed and deeply located; therefore, excessive exposure of the skull base bone was performed while resecting the cysts during surgery. During surgery, we opened the cyst and saw the cyst fluid flowing out, which was judged to be a cyst. For the cyst, we chose cyst cap removal surgery, but to reduce recurrence after the operation, we chose complete cyst resection surgery. This leads to the removal of more tissue, increasing the likelihood of exposure of the skull base bone, thus increasing the risk of SBO.

Second, although low-temperature plasma is used for cyst removal, the surgical temperature of the plasma is 60 to 80 °C which will also cause damage to the surrounding blood vessels and tissues while removing the cyst. These 2 factors lead to postoperative infection of the surgical wound, causing local destruction of the slope bone of the nasopharynx. Therefore, excessive exposure of the skull base bone and local thermal injury during surgery were the main factors for the occurrence of SBO in this patient. Previous studies have summarized the risk factors for SBO, including a history of underlying diseases such as diabetes and hypertension, a history of malignant tumors, and their treatment,^[5,8]^ Among these, a history of diabetes is the most important risk factor. Diabetes not only impairs the immune response and leads to dysfunction of T cells but also changes the local microenvironment by microvascular lesions and an increase in the pH value of body fluid, thus leading to the susceptibility of patients to base inflammation. In this case, the patient had a history of diabetes and poor blood sugar control; therefore, we believe that diabetes is a postoperative factor in this patient.

According to Muranjan et al, imaging plays a crucial role in the diagnosis of SBO. Specifically, the use of MRI with gadolinium contrast can allow for the visualization of poorly demarcated soft tissue surrounding the bone and is currently the most sensitive modality for detecting osteomyelitis. When high-resolution MRI with contrast is unavailable or contraindicated, Tc99m scans are very sensitive in detecting high osteoblastic activity and making the initial diagnosis of osteomyelitis. However, Tc99m scans will remain positive long-term even after the infection is fully treated, so gallium-67 scans are often used to follow and document resolution of the infection as the radionuclide binds acute-phase reactants in infected or inflamed tissue. In our case, because of the limitations of equipment conditions, we were unable to carry out this inspection project.

Owing to the unique anatomical location of the skull base, surgical removal of dead bone is difficult and risky. However, for SBO, the mainstream view in the literature is that surgery has a limited therapeutic effect, and the purpose of surgery is mainly to obtain deep tissue culture and biopsy under aseptic conditions for tumors.^[9,10]^ Supporters believe that SBO surgery can remove necrotic tissue, drain abscesses, and shorten the duration of use.^[11]^ For this patient, we used a treatment plan for incision and drainage of the nasopharyngeal mucosa under nasal endoscopy, mainly to clear the necrotic tissue to achieve drainage, and to take deep tissue for secretion culture. Before this, we performed multiple secretion cultures, but the results were all negative, so the treatment was empirical anti-infection treatment. Finally, the culture result of deep tissue was *Staphylococcus haemolyus* (±±), and the patient’s headache was significantly improved after changing antibiotics according to drug sensitivity. Therefore, we believe that repeated multiple secretion cultures, especially deep-tissue cultures, are necessary for nasopharyngeal infection. In this case, the pathogen responsible for the patient’s SBO was Staphylococcus hemolyticus, which is not a common pathogen. Based on experience, there is a possibility of drug resistance when using antibiotics for treatment. Drug sensitivity can guide the rational use of antibiotics and shorten the course of treatment. Different types of osteomyelitis require different medical and surgical treatment strategies. Owing to the rarity of cases, SBO has not yet formed a treatment plan, and issues such as the optimal course of treatment and the necessity and timing of surgical intervention are still controversial.^[12]^ Long-term systemic antibiotic therapy-guided pathogen culture and sensitivity testing are currently the main treatment methods for SBO.

## 4. Conclusion

In summary, SBO after endoscopic resection of nasopharyngeal cysts is extremely rare, and SBO is a rare disease with a high mortality rate. The current understanding of SBO is limited, and its diagnosis mainly relies on clinical symptoms such as headache combined with typical imaging manifestations. Diagnosis is confirmed by pathological examination results. Therefore, headache after nasal and ear surgery needs to be vigilant, and early diagnosis, early bacterial culture, full-course antibiotic anti-treatment, and minimally invasive surgical intervention are key to successful treatment. Whether nasal surgery or ear surgery is performed, excessive exposure of the normal skull base bone should be avoided during surgery to prevent SBO after surgery.

## Acknowledgments

I would like to express my gratitude to my supervisor, Prof. Ping Liu, for his great support of my project. I would also like to thank the research team for their collaboration and help in gathering data for my research project.

## Author contributions

**Conceptualization:** Wei Wang, Fang Wang.

**Data curation:** Wei Wang, Fang Wang.

**Formal analysis:** Fang Wang.

**Writing – original draft:** Wei Wang.

**Writing – review & editing:** Fang Wang, Ping Liu.
